# The regulatory mechanisms of myogenin expression in doxorubicin-treated rat cardiomyocytes

**DOI:** 10.18632/oncotarget.5338

**Published:** 2015-10-07

**Authors:** Shu-Ting Liu, Shih-Ming Huang, Ching-Liang Ho, Li-Chen Yen, Chi-Jung Huang, Wei-Shiang Lin, James Yi-Hsin Chan

**Affiliations:** ^1^ Department of Biochemistry, National Defense Medical Center, Taipei 114, Taiwan, Republic of China; ^2^ Department of Medicine, Division of Hematology/Oncology, Tri-Service General Hospital, National Defense Medical Center, Taipei 114, Taiwan, Republic of China; ^3^ Department of Medical Research, Cathay General Hospital, New Taipei City 221, Taiwan, Republic of China; ^4^ Division of Cardiology, Department of Medicine, Tri-Service General Hospital, National Defense Medical Center, Taipei City 114, Taiwan, Republic of China; ^5^ Department of Microbiology and Immunology, National Defense Medical Center, Taipei 114, Taiwan, Republic of China; ^6^ Department of Family and Community Medicine, Tri-Service General Hospital, National Defense Medical Center, Taipei 114, Taiwan, Republic of China

**Keywords:** doxorubicin, cardio-toxicity, myogenin, mRNA expression profile, gene regulation

## Abstract

Doxorubicin, an anthracycline antibiotic, has been used as an anti-neoplastic drug for almost 60 years. However, the mechanism(s) by which anthracyclines cause irreversible myocardial injury remains unclear. In order to delineate possible molecular signals involved in the myocardial toxicity, we assessed candidate genes using mRNA expression profiling in the doxorubicin-treated rat cardiomyocyte H9c2 cell line. In the study, it was confirmed that myogenin, an important transcriptional factor for muscle terminal differentiation, was significantly reduced by doxorubicin in a dose-dependent manner using both RT-PCR and western blot analyses. Also, it was identified that the doxorubicin-reduced myogenin gene level could not be rescued by most cardio-protectants. Furthermore, it was demonstrated how the signaling of the decreased myogenin expression by doxorubicin was altered at the transcriptional, post-transcriptional and translational levels. Based on these findings, a working model was proposed for relieving doxorubicin-associated myocardial toxicity by down-regulating miR-328 expression and increasing voltage-gated calcium channel β1 expression, which is a repressor of myogenin gene regulation. In summary, this study provides several lines of evidence indicating that myogenin is the target for doxorubicin-induced cardio-toxicity and a novel therapeutic strategy for doxorubicin clinical applications based on the regulatory mechanisms of myogenin expression.

## INTRODUCTION

Doxorubicin (DXR), an anthracycline antibiotic from *Streptomyces peucetius*, has been used as an anti-neoplastic drug for almost 60 years to treat several solid tumors and malignant hematological diseases [1]. Its therapeutic use to gain more wild acceptance has been hampered by toxicities, such as hematopoietic suppression, nausea, vomiting, extravasation and alopecia, yet the most feared cardio-toxicity [2–4]. While topoisomerase 2β (Top 2β) is identified as the primary mediator for the anthracycline-induced toxic effects on cardiac myocytes [5, 6], the pathogenic mechanisms responsible for anthracycline cardio-toxicity have not been fully elucidated. In a rare situation, the onset of cardio-toxicity may be delayed as many as 10–15 years after the cessation of chemotherapy.

Despite the predominant role of reactive oxidative species (ROS) in DXR-induced cardio-toxicity is certain, many of potential protectants do not appear to affect the production of free radicals but rather alter the cellular response to ROS [7–9]. Because no biomarker during DXR chemotherapy has been validated as a surrogate end point for clinically important cardiovascular disease, there is no effective approach that fully prevents or even treats DXR-associated cardio-toxicity. Presently, the measurement of cardiac enzymes or humoral factors, such as troponin I and T and B-type natriuretic peptide, are supplementary to detect early signs of DXR-induced cardio-toxicity, as they are not specific for DXR-induced damage. Thus, there remains an urgent need for methods to detect early signs of cardiac deterioration or subsequent cardiovascular events related to DXR treatment.

The use of cardio-protective drugs is an alternative approach to reduce the cardio-toxicity of DXR. However, the pharmacological and clinical attempts of cardio-protective drugs to reduce the cardio-toxicity of DXR have had little success. Thus far, dexrazoxane is the only approved drug co-administered with DXR for preventing DXR-induced cardiac damage. Its mechanisms of action involve to chelate free iron, to displace iron from DXR-iron complexes, or to prevent DXR from binding to the topisomerase 2β complex [6, 10]. Recently, many compounds including carvedilol, dexamethasone, resveratrol and berberine have been reported to possess cardio-protective activities [11–14]. But, it is still uncertain whether these drugs are useful for DXR toxicity.

While a variety of mechanisms are involved in anthracycline cardio-toxicity, it is important to develop a resembling cell-based model to study DXR-induced cardio-toxicity *in vitro*. Although H9c2 cells lack the typical organization and dense packing of adult cardio-myocytes [15, 16], they share some characteristics of differentiated cardiac cells. In the study, mRNA array profiling and H9c2 cell model were used to identify myogenin as a primary protein responded to DXR treatment. The reductive effect of DXR on myogenin expression was investigated at transcriptional, post-transcriptional, translational and post-translational levels to depict the molecular signaling of DXR toxicity. Since the decreased micro RNA-328 (miR-328) expression may play a role in DXR-induced cardio-toxicity, a potential model for the protection of DXR-induced cardio-toxicity via repression of miR-328 mediated CACNb1 (voltage-gated calcium channel β1)-dependent myogenin gene expression was proposed.

## RESULTS

### Myogenin is a target gene during myocardiac differentiation and DXR-induced cardio-toxicity

**Figure 1:**
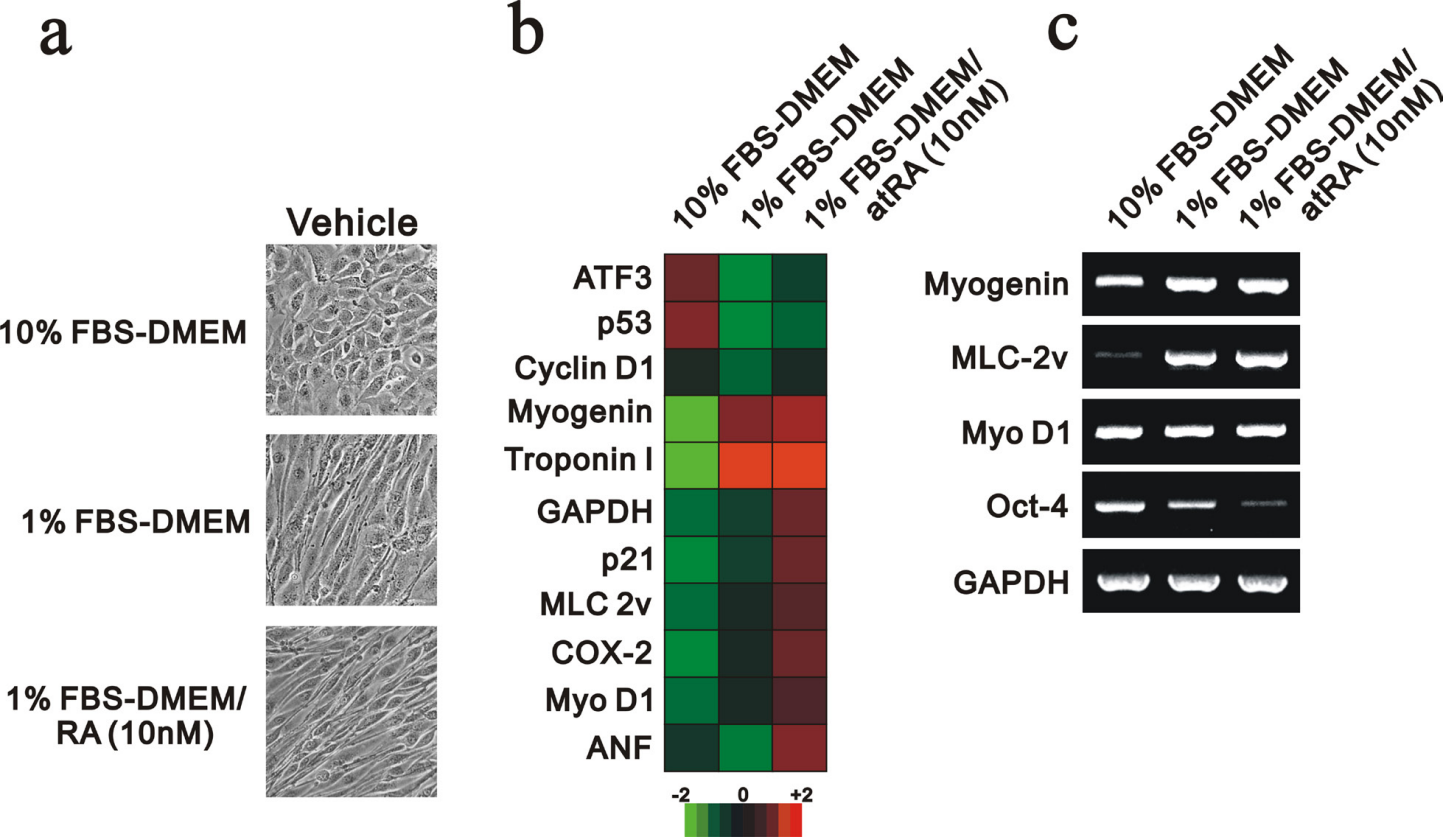
The mRNA expression profile was analyzed in differentiated H9c2 cells H9c2 cells were cultured in 10% FBS, 1% FBS and 1% FBS containing 10 nM RA. **a.** The morphology of H9c2 cells under various culture conditions was examined and recorded. **b.** Total RNAs were extracted from the indicated H9c2 cells, and mRNA expression profiling was performed. **c.** Selective genes were analyzed in the RT-PCR analysis. The results are representative of two independent experiments.

H9c2 is a subclone of the original clonal cell line derived from embryonic BD1X rat heart tissue by B. Kimes and B. Brandt and exhibits many of the properties of skeletal muscle [16]. H9c2 cells cultured in lower serum conditions and/or treated with atRA (retinoic acid) differentiate from myoblasts into myotubes [17]. Here, we first induced H9C2 cell differentiation by reducing the serum concentration to 1% or combination with atRA. We then observed the morphological changes. The cell morphology changed into a spindle shape with 1% FBS or the combined condition (Figure 1a). The differentiation status was examined in the S phase population and confirmed the significant decrease of this phase using flow-cytometry analysis (data not shown). We further collected the total RNAs and then analyzed the mRNA expression profiling using the Rat OneArray method (Phalanx Biotech Group, Taiwan, ROC). In this analysis, we found that 1% FBS significantly induced troponin I and myogenin, and atRA further induced MLC 2v and Myo D1 (Figure 1b). We reconfirmed the differentiation status of H9c2 cells, including myogenin, MLC-2v and Oct-4 [18–20], and the selected mRNAs expression profiling using the RT-PCR analysis (Figure 1c).

**Figure 2:**
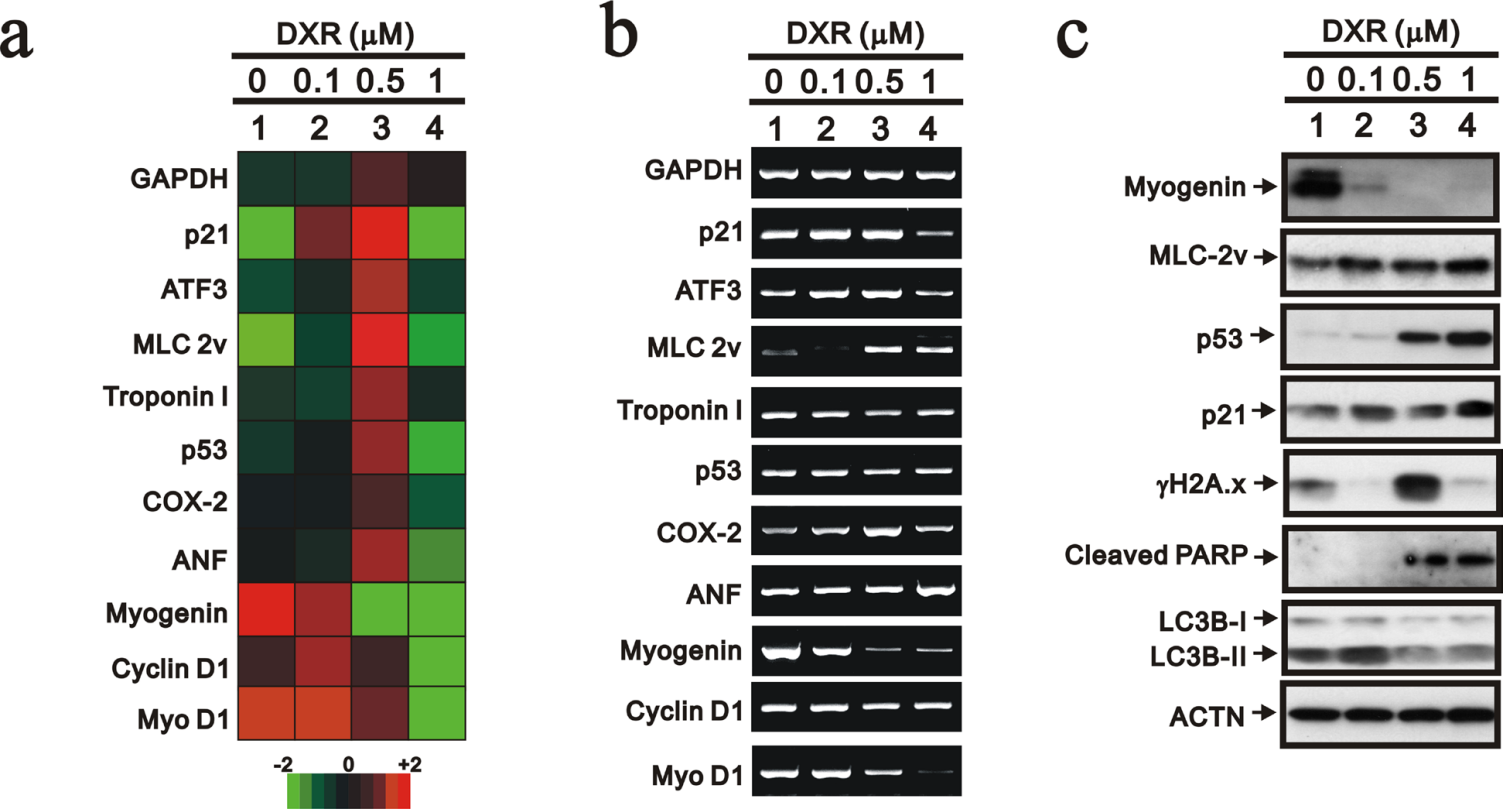
The mRNA expression profile was analyzed in DXR-treated H9c2 cells H9c2 cells were treated with the indicated amount of DXR for 16 hours. Total RNAs were extracted from the indicated H9c2 cells and then **a.** mRNA expression profiling was performed and **b.** RT-PCR analysis was performed using *GAPDH* as a loading control. **c.** Total proteins were extracted from the indicated H9c2 cells and examined by Western blotting with the indicated antibodies and ACTN (loading control). The results are representative of two independent experiments.

We further analyzed the effect of DXR on the mRNA expression levels of our selected genes, including myogenin, using the Rat OneArray method (Figure 2a). The myogenin and Myo D1 mRNAs were reduced when the concentration of DXR was increased, which was confirmed by the RT-PCR analysis (Figure 2a and 2b). The dose-dependent effects of DXR on p53, p21 and DNA damage (γH2A.x) proteins were consistent with previous studies [21]. The abundance of the myogenin protein was consistently and significantly suppressed by DXR treatment (Figure 2c). We found that the abundance of p53 proteins was increased by DXR, possibly, mediated through the ubiquitin-dependent stability [22], whereas this was inconsistent with its mRNA expression trend. The switch from autophagy (LC3II) to apoptosis (cleaved PARP) was observed for the higher DXR concentration.

### Cardio-protective drugs do not prevent the decrease in myogenin expression caused by DXR

**Figure 3:**
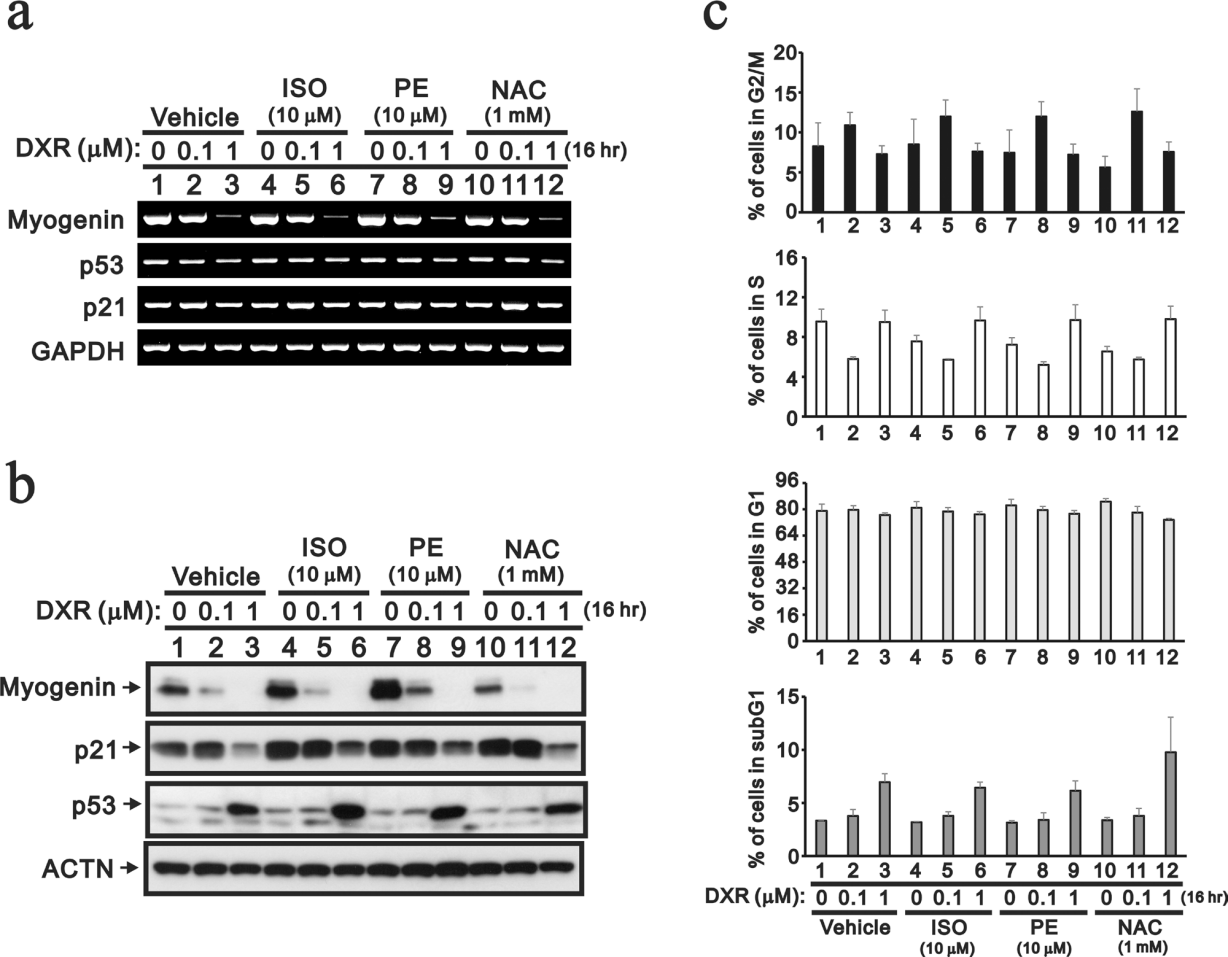
The protective effects of cardio-protective drugs were evaluated in DXR-treated H9c2 cells H9c2 cells were treated with the indicated amount of DXR with vehicle, 10 mM ISO, 10 mM PE or 1 mM NAC for 16 hours. **a.** Total RNAs were extracted from the indicated H9c2 cells and were examined by RT-PCR analysis, using *GAPDH* as a loading control. **b.** Total proteins were extracted from the indicated H9c2 cells and examined by Western blotting with antibodies against myogenin, p21, p53 and ACTN (loading control). **c.** The cells were collected and subjected to flow cytometry. The results are representative of two independent experiments.

Although the use of cardio-protective drugs is an alternative approach to reduce the cardio-toxicity of DXR, pharmacological and clinical attempts to reduce the cardio-toxicity of DXR have had little success thus far [5, 6, 12]. Isoproterenol (ISO) is a non-selective beta-adrenergic agonist used for the treatment of bradycardia and heart block. Phenylephrine (PE) is a selective α_1_-adrenergic receptor agonist used primarily as a decongestant, as an agent to dilate the pupil and as an agent to increase blood pressure. ISO and PE may relieve DXR-induced cardio-toxicity. In addition, *N*-acetyl cysteine (NAC) is a precursor in the formation of the antioxidant glutathione in the body, and its thiol group confers antioxidant effects and is able to reduce free radicals. We used NAC to examine the effect of free radicals on the decreased myogenin expression because of the primary role of ROS in DXR-induced cardio-toxicity. Here, we treated H9c2 cells with 10 μM ISO, 10 μM PE and 1 mM NAC based on our dose-course results (data not shown) and then analyzed the effects on myogenin expression. ISO and PE, but not NAC, increased the abundance of the myogenin protein, but not the mRNA (Figure 3a and 3b, compare lanes 1, 4, 7 and 10). PE prevented the decrease in myogenin protein caused by the lower concentration of DXR, whereas ISO did not (Figure 3b). NAC potentiated the effect of DXR-dependent myogenin protein repression, but enhanced the effects on the cyclin B1, ATF3, cleaved PARP and γH2A.x proteins (Figure 3b and data not shown). We observed that NAC potentiated the subG1 population induced by 1 μM DXR and reduced the population in S phase (Figure 3c). The reduction of the S phase population was also observed when H9c2 cells were treated with ISO and PE.

### The effect of DXR on myogenin expression is mediates by transcription and translation

**Figure 4:**
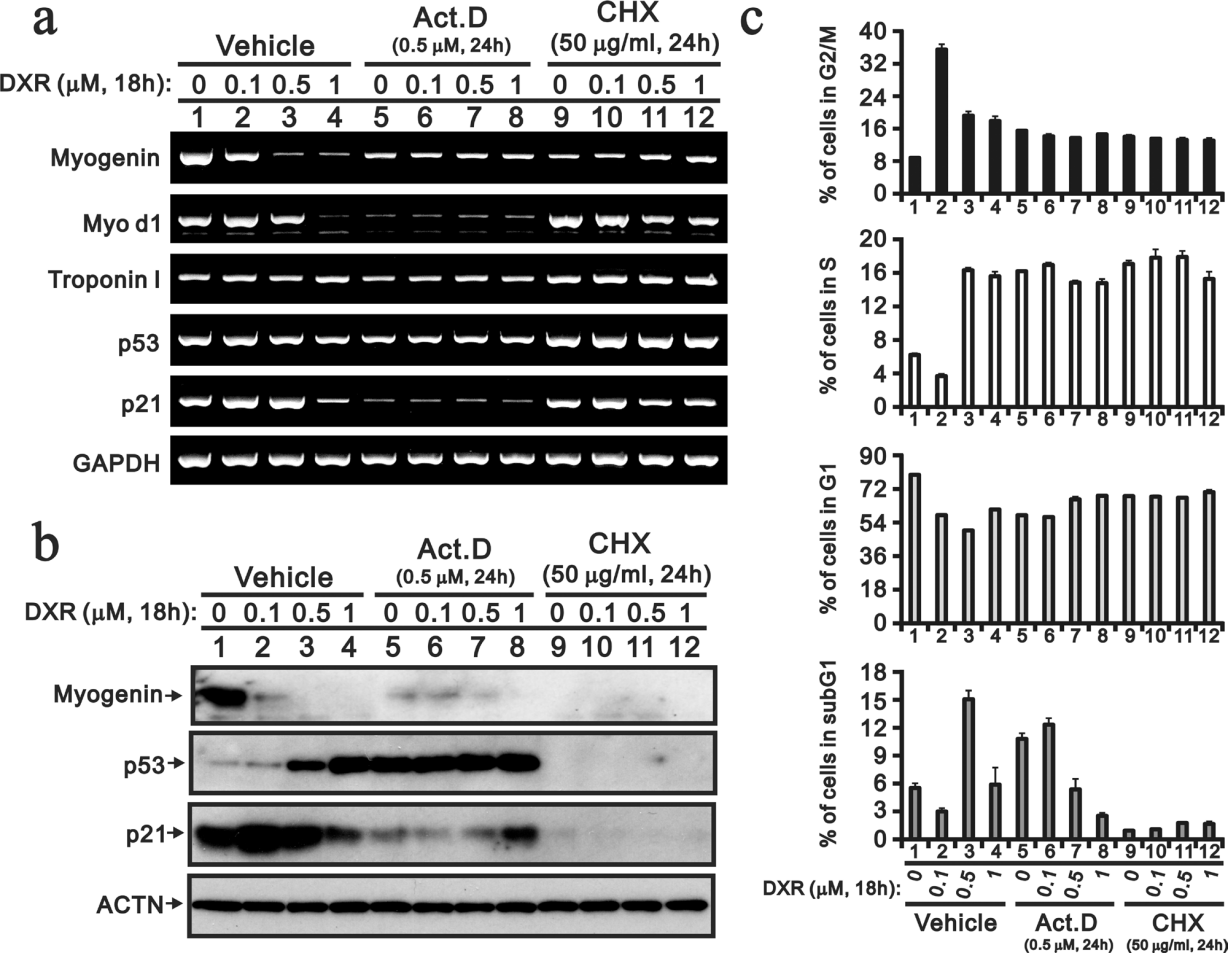
Act D and CHX prevent the DXR-induced decrease myogenin H9c2 cells were treated with the indicated amount of DXR with vehicle, 0.5 μM ActD or 50 μM NAC for 16 hours. **a.** Total RNAs were extracted from the indicated H9c2 cells and were examined with RT-PCR analysis, using *GAPDH* as a loading control. **b.** Total proteins were extracted from the indicated H9c2 cells and examined by Western blotting with antibodies again myogenin, p21, p53 and ACTN (loading control). **c.** The cells were collected and subjected to flow cytometry. The results are representative of two independent experiments.

Act D inhibits transcription because it binds DNA at the transcription initiation complex and prevents the elongation of the RNA chain by RNA polymerase [23]. CHX is a de novo protein biosynthesis inhibitor and exerts its effect by interfering with the translocation step in protein synthesis, thus blocking translational elongation [24]. Act D and CHX were used to elucidate the effect of DXR on the transcriptional stage or/and translational stage (Figure 4). Here, Act D was useful to determine the mRNA stability of the selected genes. The *p53* and control *GAPDH* mRNAs were stable; however, the majority of the mRNAs were labile, particularly *MyoD1* and *p21* (Figure 4a). The suppression effect of DXR on *myogenin* mRNA was alleviated in with Act D treatment. The Act D induction of p53 proteins was observed, and other protein expression levels were consistent with the effect of Act D at the transcriptional stage (Figure 4b). Higher concentrations of DXR also had similar Act D effects on the *p53* gene and protein. The decrease of the *myogenin* gene and protein by DXR were prevented by Act D, suggesting that the regulatory mechanism is primarily at the transcriptional stage. We found that the myogenin protein was unstable. The p53 and p21 proteins were also unstable when the H9c2 cells were treated with CHX. Our flow cytometry analysis was consistent with the so-called DXR dose effect, such that a lower dose (0.1 μg) induced G2/M and a higher dose (0.5 μg) induced apoptosis (Figure 4c, vehicle). For the combination of DXR with Act D or CHX, higher amounts of DXR predominantly determined the cell cycle at the G1, S and G2/M phases (Figure 4c). Based on the subG1 population, Act D induced apoptosis and suppressed higher amounts of DXR-induced apoptosis. CHX reduced apoptosis in H9c2 cells, with or without the addition of DXR (Figure 4c).

**Figure 5:**
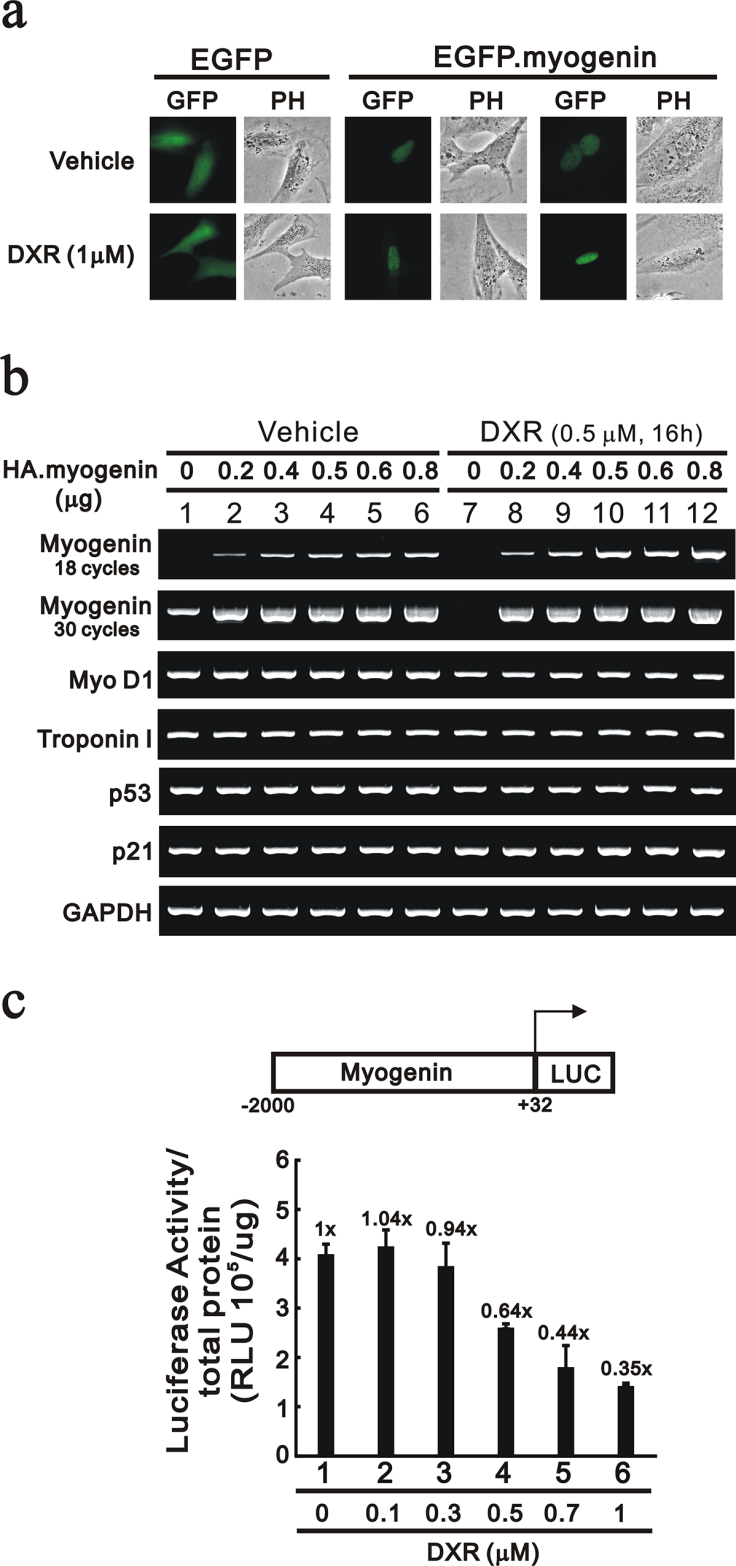
DXR suppresses myogenin promoter activity in H9c2 cells **a.** H9c2 cells were transiently transfected with 0.5 μg pEGFP vector or pEGFP.myogenin. Approximately 10 representative transfected H9c2 cells were examined under a fluorescence microscope. **b.** H9c2 cells were transiently transfected with 0.8 μg pSG5.HA vector or the indicated amount of pSG5.HA.myogenin and were treated with the indicated amount of DXR for 16 hours. Total RNAs were extracted and then were examined by RT-PCR analysis, using *GAPDH* as a loading control. **c.** H9c2 cells were transiently transfected with 0.3 μg of the *myogenin* promoter report plasmid, pGL3.myogenin(−2000/+32)-LUC, for 3 hours and were treated with the indicated amount of DXR for 16 hours. The luciferase activity was normalized by total proteins. The numbers above the columns indicate the luciferase activity relative to an index of 1 for the reporter vector alone. The results (b and c) are representative of two independent experiments.

We constructed an expression vector for myogenin to investigate the regulatory mechanism of DXR. First, our data demonstrated that myogenin localized to the nucleus via the EGFP expression vector and DXR had no apparent effect on its subcellular localization (Figure 5a). Next, we transiently transfected the indicated serial amount of HA-fused myogenin and examined the decreased effect of DXR on the exogenous *myogenin* mRNA and protein in H9c2 cells. Our data demonstrated that exogenous *myogenin* mRNAs were dose-dependent, and DXR selectively depleted the endogenous *myogenin* mRNA, but not the exogenous *myogenin* mRNA (Figure 5b, compare lanes 1 and 7 in 18 cycles of PCR). Therefore, we further determined whether DXR directly suppresses *myogenin* promoter activity. Our data show that DXR did repress the *myogenin* promoter activity in a concentration-dependent manner (Figure 5c).

**Figure 6:**
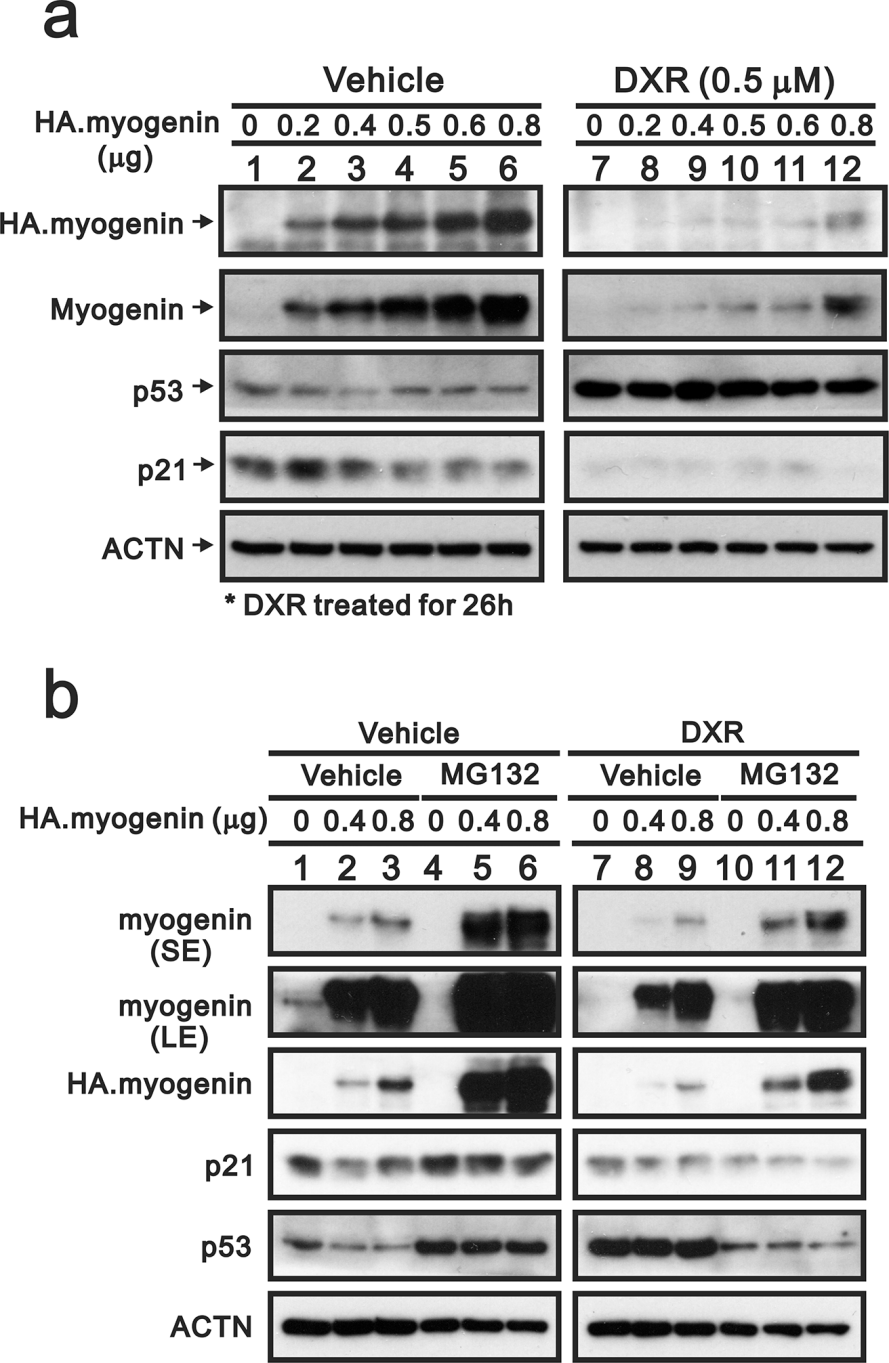
MG132 prevents the decrease of myogenin induced by DXR in H9c2 cells **a.** H9c2 cells were transiently transfected with 0.8 μg pSG5.HA vector or the indicated amount of pSG5.HA.myogenin and were treated with vehicle or 0.5 μM DXR for 16 hours. **b.** H9c2 cells were transiently transfected with 0.8 μg pSG5.HA vector or the indicated amount of pSG5.HA.myogenin and were pretreated with MG132 for 4 hours. H9c2 cells were further treated with vehicle or 0.5 μM DXR for 16 hours. Total proteins were extracted from the indicated H9c2 cells and examined by Western blot with antibodies against HA, myogenin, p21, p53 and ACTN (loading control). The results are representative of two independent experiments.

In contrast to the effect on the transcriptional level (Figure 5b), DXR depleted the endogenous and exogenous myogenin proteins (Figure 6a). We further verified the protein degradation pathway of DXR-induced myogenin using the proteasome inhibitor, MG132, in H9c2 cells. The myogenin and p53 proteins were more stable in the pretreated MG132-H9c2 cells (Figure 6b, compare lanes 4–6 to lanes 1–3). DXR significantly decreased the myogenin proteins, but MG132 partially prevented this decrease (Figure 6b, compare lanes 4–6 to 10–12), suggesting that DXR may down-regulate myogenin proteins via mechanisms other than the proteasome-degradation pathway. In addition, the stabilization of p53 proteins was observed for the MG132 and DXR treatment conditions, whereas this stability was disrupted for the combination of MG132 and DXR in H9c2 cells (Figure 6b).

### Twist, HuR and miR-186 are not involved in the effect of DXR on myogenin expression

**Figure 7:**
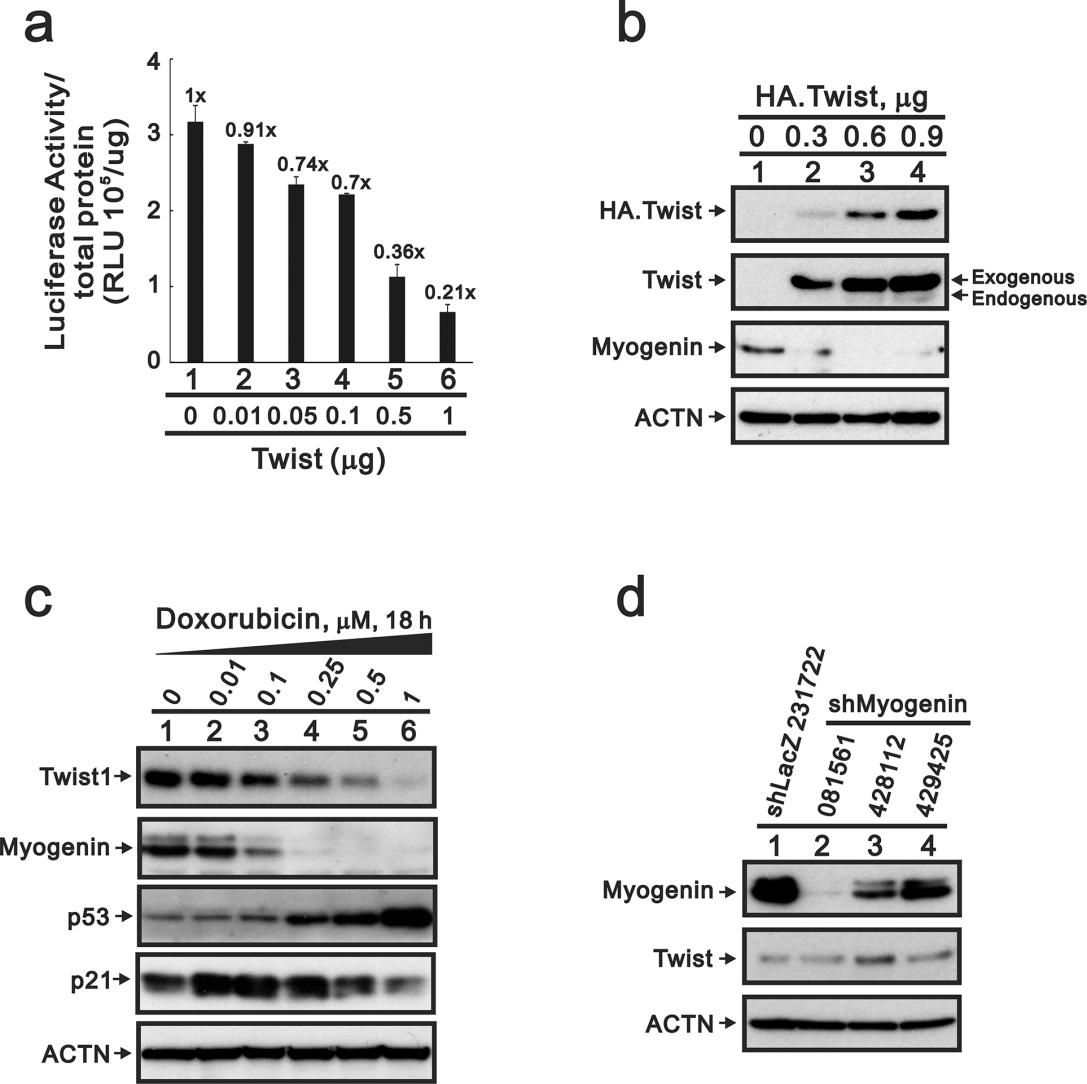
The suppression of myogenin expression by Twist does not occur in DXR-treated H9c2 cells **a.** H9c2 cells were transiently transfected with 0.3 μg of the *myogenin* promoter report plasmid, pGL3.myogenin (−2000/+32)-LUC, and the indicated amount pSG5.HA.Twist. The luciferase activity was normalized by total proteins. The numbers above the columns indicate the luciferase activity relative to an index of 1 for the reporter vector alone. **b.** H9c2 cells were transiently transfected with 0.9 μg pSG5.HA vector or the indicated amount of pSG5.HA.Twist. **c.** H9c2 cells were treated with the indicated amount of DXR for 16 hours. Total proteins were extracted and examined by Western blotting with antibodies against HA, Twist, myogenin, p53, p21 and ACTN as a loading control. **d.** Total proteins were extracted from shLacZ (control) or three clones of shMyogenin H9c2 cells and examined by Western blot with antibodies against myogenin, Twist and ACTN (loading control). The results (a–c) are representative of two independent experiments.

A previous study demonstrated that transcription factor, Twist, down-regulates myogenin gene expression [25]. Our myogenin promoter reporter assays were consistent with the suppressive effect of Twist proteins on this promoter activity in H9c2 cells (Figure 7a). Our western blotting data further demonstrated that overexpressing Twist decreased the level of myogenin protein and its effect on the *myogenin* promoter activity (Figure 7b). If Twist proteins are involved in the repression of myogenin in the presence of DXR, then the expression should increase with DXR treatment. Our data showed that the level of the Twist protein was decreased after the depletion of myogenin proteins in DXR-treated H9c2 cells (Figure 7c, lane 4), and the p53 protein was induced at this point. We knocked-down the *myogenin* gene to examine the relationship between myogenin and Twist. Our data demonstrated that the endogenous myogenin protein reduced the abundance of the Twist protein (Figure 7d).

**Figure 8:**
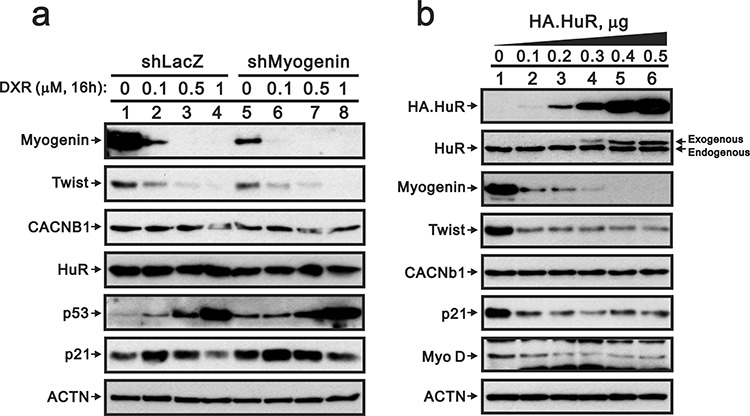
Overexpressing HuR suppresses myogenin, Twist, p21 and MyoD in H9c2 cells **a.** ShLacZ (control) or shMyogenin H9c2 cells were treated with the indicated amount of DXR for 16 hours. **b.** H9c2 cells were transiently transfected with 0.5 μg pSG5.HA vector or the indicated amount of pSG5.HA.HuR. Total proteins were extracted and examined by Western blot with antibodies against HA, HuR, Twist, myogenin, CACNb1, p21, MyoD and ACTN (loading control). The results are representative of two independent experiments.

Because Twist proteins do not play an important role in the decreased myogenin expression caused by DXR, we further examined other potential candidates involved in this process. MiR-186, HuR and CACNb1 may mediate myogenin expression through different pathways [26–28]. Here, we first analyzed the miR profile using the rat miR OneArray kit and found 22 up-regulated and 17 down-regulated miRNAs for 1 μM DXR compared to the vehicle treatment (Table 1). Unfortunately, we did not identify miR-186. The 22 up-regulated miRNAs were not involved in myogenin mRNA stability or translation. Next, we examined the effect of DXR on HuR protein expression, which remained constant levels (Figure 8a, left panel). In contrast, exogenously overexpressed HuR significantly decreased the abundance of the myogenin protein (Figure 8b, compare lanes 1 and 2). Recently, Dr. Delbono's laboratory demonstrated that CACNb1 suppresses *myogenin* gene expression in mouse progenitor cells [28]. However, we demonstrated that DXR or exogenously overexpressed HuR proteins did not increase the abundance of the CACNb1 proteins.

**Table 1:** miRs modulated in DXR-treated H9c2 cells

DXR, μM	up-regulated miRs	down-regulated miRs
0.1	rno-miR-133b-3p rno-miR-3575	rno-miR-674-5p
0.5	rno-miR-18a-3p rno-miR-505-5p rno-miR-204-3p rno-miR-92a-2-5p	rno-let-7e-5p rno-miR-125b-2-3p rno-miR-674-5p rno-miR-298-5p rno-miR-328a-5p rno-miR-3558-3p rno-miR-3473
1	rno-miR-184 rno-miR-291a-5p rno-miR-294 rno-miR-194-3p rno-miR-297 rno-miR-466c-5p rno-miR-30c-1-3p rno-miR-760-3p rno-miR-542-5p rno-miR-128–2-5p rno-miR-210-3p rno-miR-320-3p rno-miR-326-5p rno-miR-505-5p rno-miR-675-5p rno-miR-770-3p rno-miR-204-3p rno-miR-3543 rno-miR-3573-5p rno-miR-3575 rno-miR-541-3p rno-miR-92a-2-5p	rno-miR-125b-5p rno-let-7e-5p rno-miR-22-3p rno-miR-181a-5p rno-miR-125b-2-3p rno-miR-24-3p rno-miR-29a-3p rno-miR-21-5p rno-miR-347 rno-miR-298-5p rno-miR-328a-5p rno-miR-351-5p rno-miR-375-5p rno-miR-758-5p rno-miR-3558-3p rno-miR-3473 “rno-miR-129-1-3p, rno-miR-129-2-3p”

## DISCUSSION

Previous studies support the effector role of many myogenic transcriptional factors, including Myo D1, in DXR-induced cardio-toxicity [29, 30]. However, these myogenic transcriptional factors are mainly involved during the early stage. Myo D1 and myogenin have distinct regulatory roles at a similar set of target genes. Myogenin activity is a pivot point for the irreversible commitment to terminal differentiation. For these late genes, myogenin does not bind efficiently without Myo D1; however, transcriptional activation requires the combined activity of Myo D1 and myogenin. Therefore, the role of myogenin in mediating terminal differentiation is, in part, to enhance the expression of a subset of genes previously initiated by Myo D1. Recently, studies from Dr. Phylactou's laboratory reveal an important role for myogenin in maintaining terminal muscle cell differentiation and suggest a novel mechanism by which muscle cells may be reactivated through its down-regulation [31]. Here, our study is the first to demonstrate that DXR modulates the *myogenin* gene mediated at the transcriptional, post-transcriptional, translational and post-translational levels. In addition, several miRs were also modulated by DXR based on our miRNA analysis profile (Table 1), such as miR-328. A study by Dr. Yang's laboratory revealed that CACNb1 was the target of miR-328, which was suppressed by DXR [32], and Dr. Delbono's laboratory demonstrated that CACNb1 suppresses *myogenin* gene expression in mouse progenitor cells [28]. We subsequently proposed the possible involvement of CACNb1 via the suppression of miR-328 expression to down-regulate *myogenin* gene expression in rat cardio-myocytes. The functional role of HuR in myogenin regulation will be further addressed because of the significant reduction of the myogenin protein by exogenous HuR (Figure 8b). This further understanding of the target of DXR-induced cardio-toxicity will help us to identify cardio-protective agents.

The mechanism of action of DXR is dependent on its concentration and remains a matter of debate [33, 34]. Our current results in cardiomyocyte H9c2 cells were consistent with previous studies demonstrating that at the senescence-inducing concentration (0.1 μM), DXR induced more DNA damage, as measured by phosphorylation of γ-H2A.X, (0.5 μM) than at the apoptosis-inducing concentration (1 μM) in human cervical carcinoma cells or other cells. The expression level of p21 correlates with the activation by p53 at low DXR concentrations, but not at high concentrations. ROS has a predominant role in DXR-induced cardio-toxicity and the only FDA-approved free iron chelator is dexrazoxane [35]. NAC did not prevent the decrease in myogenin gene and protein expression caused by DXR in this study. Many studies suggest that ROS and iron become toxic as a result of reactions that extend beyond canonical oxidative damage [36, 37]. Therefore, it is to characterize the relationship between ROS and myogenin expression upon DXR treatment.

Myogenin is essential during differentiation [20, 29]. Downregulation of endogenous *myogenin* gene expression in muscle cells can lead to the reversal of muscle cell differentiation and the creation of mononucleated cells. MyoD and Myf5 are unable to substitute for the function of myogenin during differentiation [30]. There are many factors involved in the regulation of *myogenin* gene expression, such as HuR, Twist, CACNb1, miR-186 and miR-328 [25–28, 32]. The mechanisms involved in myogenin reduction during heart failure are not completely understood. In cell culture, *myogenin* expression is suppressed in differentiating myocytes treated with tumor necrosis factor-α (TNF-α), a cytokine that commonly increases during heart failure. Additionally, TNF-α is a major inducer of chronic inflammation and ROS and is abundant under conditions of chronic inflammation. A recent study by Dr. Baba's laboratory demonstrated that miR-328 expression was markedly suppressed in gastric cancer cells treated with H_2_O_2_, but not TNF-α [38]. Combined with these findings, the suppression of myogenin by TNF-αis either independent of the miR-328-CACNb1 signaling in myocytes or the selectivity of the miR-328 target depends on the cell-type. However, the suppression of miR-328 by ROS is not the primary mechanism because NAC did not affect the abundance of myogenin in this study.

DXR induced cardiomyocyte atrophy, which was alleviated by the proteasome inhibitor, MG132, indicating a role for the ubiquitin-proteasome system in DXR-induced atrophy [3, 39]. Our data indicate that MG132 prevent the DXR induced decrease in myogenin protein in H9c2 cells, suggesting that myogenin may play a role in DXR-induced atrophy. Recent studies demonstrate that Twist and miR-168 both suppress *myogenin* gene expression [25, 27]. In this study, the involvement of Twist and miR-168 in the DXR-induced myogenin decrease in H9c2 cells was ruled out. However, Dr. Yang's laboratory showed that CACNb1 is the target of miR-328, and Dr. Delbono's laboratory further demonstrated that CACNb1 suppresses *myogenin* gene expression mediated through the binding of CACNb1 to the *myogenin* promoter in mouse progenitor cells [28, 32]. In addition, a recent study demonstrates modulation of the L-type calcium channel expression during retinoic acid-induced differentiation of H9c2 cells [17]. Therefore, we will have the chance to correlate the linkage of miR-328-dependent CACNB1 signaling with the depletion of myogenin proteins to determine the mechanism of DXR-induced cardio-toxicity in the future.

The discovery of widespread functions of miRs has increased the complexity of gene-regulatory processes and networks, in both the cardiovascular system and cardiovascular diseases [40]. Mechanistically, miRs regulate gene expression at the post-transcriptional level at the 3′-UTR by inhibiting the translation of protein from the mRNA or by promoting the degradation of mRNA. One of the challenges for the future use of miR-based therapies arises from the fact that many miRs modulate multiple target genes (100 or more) involved in multiple cellular processes. Although the repressive effects of any individual miR on a single target might be subtle, the combinatorial effects of a miR on multiple mRNA targets within a regulatory network can profoundly change the output of a pathway. Using the TargetScan website to predict human miR-328 targets, human myogenin was identified as a potential target; however, rat myogenin is not its target because of its distinctive 3′-UTR sequences, GGGCCAA and GGGCAA (data not shown). Dr. Yang demonstrated that CACNB1 was a target for miR-328, but there was no supporting data from the TargetScan analysis. Here, we propose that the DXR-induced decrease of myogenin is mediated through transcriptional repression by CACNb1, which is induced by the downregulation of miR-328 by DXR in rat cardiomyocytes. It is important to elucidate the detailed regulatory mechanism of miR-328 and its targets under various pathophysiological conditions, such as atrial fibrillation.

In summary, the results of this study identify myogenin as a new DXR-induced cardio-toxicity target and indicate that it is transcriptionally, post-transcriptionally, translationally, and post-translationally regulated. Furthermore, HuR and miR-328-dependent CACNB1 signaling are potentially involved in the depletion of the myogenin gene and protein by DXR in rat cardiomyocytes. Therefore, the regulation of *myogenin* expression may represent a novel therapeutic target for DXR clinical applications.

## MATERIALS AND METHODS

### Cell culture and chemicals

H9c2 cells were cultured in Dulbecco's modified Eagle's medium supplemented with 10% or 1% fetal bovine serum and 1% penicillin-streptomycin (Invitrogen, CA, USA). All-trans retinoic acid (atRA), DXR, actinomycin D (Act D), cycloheximide (CHX), isoproterenol (ISO), phenylephrine (PE) and *N*-acetyl cysteine (NAC) were purchased from Sigma-Aldrich (MO, USA).

### Messenger RNA and miRNA expression profiling

Total RNAs were extracted from H9c2 cells using the TRIsure (Bioline Reagents, London, UK) reagent and were was sent to the Phalanx Biotech Group (HsinChu, Taiwan, ROC) for their mRNA expression profiling service using the Rat OneArray method and for their miRNA expression profiling service using the Mouse and Rat miRNA OneArray. The RNA quantity and purity was assessed using a NanoDrop ND-1000. The pass criteria for the absorbance ratios are established at A260/A280 ≥ 1.8 and A260/A230 ≥ 1.5, indicating acceptable RNA purity. The RIN values were ascertained using the Agilent RNA 6000 Nano assay to determine the RNA integrity. The pass criteria for the RIN value were established at ≥6, indicating acceptable RNA integrity. gDNA contamination was evaluated by gel electrophoresis. The data analysis was performed with the Rosetta Resolver^®^ System (Rosetta Biosoftware, WA, USA).

### Plasmids, transfection and luciferase reporter assay

*Myogenin* was constructed by inserting the full-length PCR fragments into the pSG5.HA and pEGFP vectors via the *EcoR*I-*Xho*I and *EcoR*I-*Sal*I restriction sites, respectively. *Twist* and *HuR* were constructed by inserting the full-length PCR fragments into the pSG5.HA vector via the *EcoR*I-*Xho*I restriction sites. The reporter *myogenin*(−2000/+33)-LUC was constructed by inserting the appropriate PCR fragments into the pGL3-LUC reporter plasmid via the *Kpn*I-*Xho*I restriction sites. The cells in each well (6- or 24-well plate) were transfected with jetPEI (PolyPlus-transfection, Illkirch, France) according to the manufacturer's protocol. The total DNA was adjusted to 2.0 μg (6-well) or 1.0 μg (24-well) with the addition of empty pSG5.HA vector. Luciferase assays were performed with the Promega Luciferase Assay kit (Promega, MI, USA), and the measurement is expressed numerically as relative light units (RLU). We normalized these reporter assays with individual total proteins because the renilla internal control system was downregulated by DXR and Twist in H9c2 cells. The luciferase activities are shown as the mean and deviation from the mean of two transfected sets as previously described [41].

### Western blot analysis

The cell lysates were prepared in lysis buffer (100 mM Tris-HCl, pH 8.0, 150 mM NaCl, 0.1% SDS and 1% Triton X-100) at 4°C. The extracts were separated by SDS-PAGE, transferred onto a polyvinylidine difluoride membrane (Millipore, MA, USA) and detected using antibodies against PARP, LC3 (Cell Signaling Technology, MA, USA), α-actinin (ACTN), p53, p21, MLC-2v, H3P, HuR, Twist, CACNb1 (Santa Cruz Biotechnology, CA, USA), γH2A.X, myogenin (Epitomics, CA, USA) and hemagglutinin (HA) (3F10, Hoffmann-La Roche, Basel, Switzerland).

### Reverse transcription-polymerase chain reaction (RT-PCR)

Total RNA was isolated using the TRIsure reagent according to the manufacturer's instructions. One microgram of total RNA was subjected to reverse transcription using MMLV reverse transcriptase for 60 min at 37°C (Epicentre Biotechnologies, MI, USA). The PCR reactions were assayed on a Veriti^™^ Simpli Thermal Cycler (Applied Biosystems, CA, USA). All PCR primer sequences are shown in Table 2.

**Table 2:** PCR primers used in this study

Gene name	Primer sequence (5′ → 3′)
***myogenin***	Forward: 5′-CTA CCT TCC TGT CCA CCT TC-3′ Reverse: 5′-CTC CAG TGC ATT GCC CCA CT-3′
***MLC 2v***	Forward: 5′-CTC CAA CGT GTT CTC CAT G-3′ Reverse: 5′-AGT CCT TCT CTT CTC CGT GGG-3′
***Troponin I***	Forward: 5′-GCA AAA GTC ACC AAG AAC ATC-3′ Reverse: 5′-GCG CCA GTC TCC CAC CTC CCG G-3′
***Myo D1***	Forward: 5′-CAG CGG GCA CCA CCA G-3′ Reverse: 5′-ATG CTG GAC AGG CAG TC-3′
***p53***	Forward: 5′-GCA ACT ATG GCT TCC ACC TG-3′ Reverse: 5′-CAC GAA CCT CAA AGC TGT CC-3′
***p21***	Forward: 5′-GCT GTC TCC AGG AGG CCC G-3′ Reverse: 5′-GCT GGT CTG CCT CCG TTT TCG-3′
***cyclin D1***	Forward: 5′-ATG GAA CAC CAG CTC CTG TG-3′ Reverse: 5′-CTT AGA GGC CAC GAA CAT GC-3′
***ATF3***	Forward: 5′-GAG GAT TTT GCT AAC CTG AC-3′ Reverse: 5′-TAG CTC TGC AAT GTT CCT TC-3′
***GAPDH***	Forward: 5′-CTT CAT TGA CCT CAA CTA C-3′ Reverse: 5′-GCC ATC CAC AGT CTT CTG-3′
***COX-2***	Forward: 5′- GTC TCT CAT CTG CAA TAA TGT G-3′ Reverse: 5′- ATC TGT GTG GGT ACA AAT TTG-3′
***ANF***	Forward: 5′-CTG CTA GAC CAC CTG GAG GA-3′ Reverse: 5′- AAG CTG TTG CAG CCT AGT CC-3′
***Nkx2.5***	Forward: 5′-GTG CTG AAG CTC ACG TCC AC-3′ Reverse: 5′-CGA CGC CAA AGT TCA CGA AG-3′
***Oct4***	Forward: 5′-GAG AAC CGT GTG AGG TGG AAC-3′ Reverse: 5′-CCT CAG GAA AAG GGA CCG AG-3′

### Fluorescence-activated cell sorting (FACS) analysis

For cell cycle evaluation, the cells were treated using the same procedure as for the proliferation experiments, washed with ice-cold PBS and incubated with PI solution (0.05% mg/ml in PBS, 0.1% Triton X-100 and 0.01% RNase) for 15 min at room temperature in the dark. FACS analysis was based on the measurement of the DNA content of nuclei labeled with propidium iodide (PI). The cells were then subjected to FACS, and cell cycle analysis was performed using a FACSCalibur flow cytometer and the Cell Quest Pro software (BD Biosciences, CA, USA) as previously described [21].

### Gene silencing

The shRNA lenti-viral vectors targeting mouse *myogenin* (Clone ID: #1:TRCN0000081561 and #2: TRCN0000428112 and #3: TRCN0000429425) for the screening of rat myogenin and a pLKO control lenti-viral vector (Clone ID: TRCN0000231722) were purchased from the National RNAi Core Facility, Academia Sinica, Taiwan. Cells infected with the lenti-virus containing target gene shRNAs were selected in 2 μg/ml puromycin. Pooled populations of knockdown cells were used for the experiments.

## Abbreviations

DXR: Doxorubicin
Top 2β: topoisomerase 2β
ROS: reactive oxidative species
miR-328: micro RNA-328
CACNb1: voltage-gated calcium channel β1
atRA: all-trans retinoic acid
Act D: actinomycin D
CHX: cycloheximide
ISO: isoproterenol
PE: phenylephrine
NAC: N-acetyl cysteine
RLU: relative light units
ACTN: α-actinin
HA: hemagglutinin
FACS: fluorescence-activated cell sorting
PI: propidium iodide.
